# Stepwise micro-CT analysis of sample processing effects on the 3D morphology of human intervertebral discs

**DOI:** 10.1016/j.ocarto.2026.100857

**Published:** 2026-08-12

**Authors:** Raluca-Ana-Maria Barna, Federica Orellana, Marie-Rosa Fasser, Simone Baffelli, Daniel Valent, Jonas Widmer, Annapaola Parrilli

**Affiliations:** a Center for X-Ray Analytics, Empa, Dübendorf, Switzerland; b Faculty of Medicine, University of Bern, Bern, Switzerland; c Spine Biomechanics, Department of Orthopedic Surgery, Balgrist University Hospital, University of Zurich, Zurich, Switzerland; d AO Research Institute Davos, Davos, Switzerland; e Scientific IT, Empa, Dübendorf, Switzerland

**Keywords:** Contrast-enhanced micro-CT, Soft tissue imaging, Intervertebral disc, Formalin fixation, Lugol’s iodine staining, Sample preparation artifacts

## Abstract

**Objective:**

Structural alterations of the intervertebral disc (IVD) contribute to low back pain and affect spinal biomechanics. Three-dimensional imaging of disc soft tissues requires sample preparation steps that may introduce artifacts and alter native morphology. This study presents a stepwise contrast-enhanced micro-CT evaluation of how sample processing affects the 3D morphology of human IVDs, aiming to quantify changes in volumetric parameters and radiodensity across preparation stages.

**Design:**

Human IVDs were imaged using micro-CT at four sequential stages: (i) frozen, (ii) thawed, (iii) after formalin fixation, and (iv) after Lugol iodine contrast staining. Qualitative and quantitative analyses assessed changes in tissue volume, lesions, rim tears, calcifications, and radiodensity across stages. Thawed samples served as the volumetric reference.

**Results:**

Frozen discs preserved annulus fibrosus lamellar architecture but showed reduced volume relative to the thawed state and low radiodensity. Formalin fixation induced the largest morphological changes, increasing mean disc volume by 21.5% and causing deformation associated with nucleus pulposus swelling. Subsequent staining caused partial shrinkage relative to the fixed condition but markedly increased X-ray attenuation (148.6% vs frozen), enabling improved visualization of internal disc microstructures. Internal lesions remained detectable across preparation stages, whereas calcifications appeared reduced after staining due to decreased contrast with surrounding soft tissue.

**Conclusions:**

Each preparation step introduces distinct and quantifiable effects on IVD morphology and imaging contrast. These findings provide a methodological reference for optimizing contrast-enhanced micro-CT workflows and for distinguishing biological features from preparation-induced artifacts in IVD research.

## 1 Introduction

Low back pain is a leading cause of disability worldwide, and its burden is expected to rise substantially as populations age, with projections estimating up to 843 million prevalent cases by 2050 [1,2]. Intervertebral disc (IVD) degeneration is highly prevalent in the adult population, increases markedly with age, and has been associated with low back pain, particularly when affecting the lumbar spine [3].

The IVD is a fibrocartilaginous tissue composed of a gel-like hydrated structure, the nucleus pulposus (NP), surrounded externally by the annulus fibrosus (AF), a thick collagenous ring, and superiorly and inferiorly by cartilage endplates (CEPs). Its primary functions include transmitting loads through the vertebral column, providing flexibility, and enabling movements like bending, flexion, and torsion of the spine [4,5]. These mechanical roles are supported by collagen type I and II fibrils, which offer tensile strength [6,7] and negatively charged proteoglycans, which help to retain water content and, thus, maintain the hydration of the tissue [8,9].

The degeneration of the IVD, characterized by reduced disc height, loss of elasticity, increased fibrosis, and altered spinal mechanics, is closely associated with low back pain [[9], [10], [11]]. These degenerative and age-related changes in the IVD primarily manifest as significant morphological alterations, including radial, concentric, and rim tears, delamination, and tissue breakdown in the form of microlesions and fissures [12].

Depending on clinical presentation and underlying pathology, treatment options range from non-surgical approaches, including physiotherapy, exercise, analgesics, anti-inflammatory drugs, and selected spinal injections, to surgical procedures such as discectomy or spinal fusion when clinically indicated [13].

The diagnosis of intervertebral disc degeneration commonly relies on imaging techniques such as radiography, MRI, and CT [14]. MRI is the preferred modality for differentiating the different types of disc herniation, whereas radiographs reveal intervertebral space narrowing, and CT can detect specific signs of degeneration, such as calcifications [11]. Although these imaging modalities are essential for clinical assessment, investigating the microstructure of the IVD and its alterations during degeneration or other processes requires higher-resolution imaging approaches.

Previous studies have employed two-dimensional (2D) microscopy to visualize the lamellar structure and the orientation of collagen fibers in the AF [15,16]. Given that the microstructure and tissue organization underpin the biomechanical functions of the IVD, 2D microscopy has also been applied for modeling the inter-lamellar shear resistance and its role in the AF biomechanical behavior [17]. However, these 2D methods are inherently destructive, relying on tissue sectioning and a layer-by-layer approach, which limits their ability to preserve the native microstructure for subsequent analysis.

Regarding three-dimensional (3D) techniques, recent imaging methods have been employed to non-destructively analyze the 3D microstructure of the IVD, both qualitatively and quantitatively. For instance, MRI diffusion tensor imaging has been utilized to study the structure and orientation of collagen fibers in the human AF while preserving the 3D integrity of the tissue [18]. X-ray CT, when used at micrometric resolution (micro-CT), offers a powerful non-destructive imaging method for visualizing the 3D structure of the entire IVD with high resolution. contrast agents, such as Phosphomolybdic Acid, Phosphotungstic Acid, or iodine-based solutions, are necessary to stain the IVDs, enhancing X-ray absorption in soft tissues and thereby improving the visibility and contrast of their 3D microstructure during imaging [19,20]. The significant advantages of the micro-CT technique lie in its ability to generate detailed, high-resolution 3D models and to quantify various geometrical parameters without compromising tissue integrity [21,22]. These advantages have enabled quantitative analyses in animal models of the spine, such as the quantification of AF, NP, and notochord volumes in murine discs during post-natal development [23] and the investigation of bovine IVD microstructure [24].

However, a comprehensive 3D characterization of the entire human IVD microstructure remains limited.

In this study, we employed high-resolution contrast-enhanced micro-CT to visualize the microstructure of human IVDs *ex vivo*. Since contrast-enhanced micro-CT requires multiple stages of sample processing, the study aimed to quantify and compare changes in specific volumetric parameters and radiodensity. In addition, we evaluated the extent of potential deformations induced by the different stages of sample processing. Specifically, we quantified and compared morphological changes caused by freezing, formalin fixation, and iodine-based staining.

## 2 Method

### 2.1 Tissue preparation and staining procedure

**Fig. 1:**
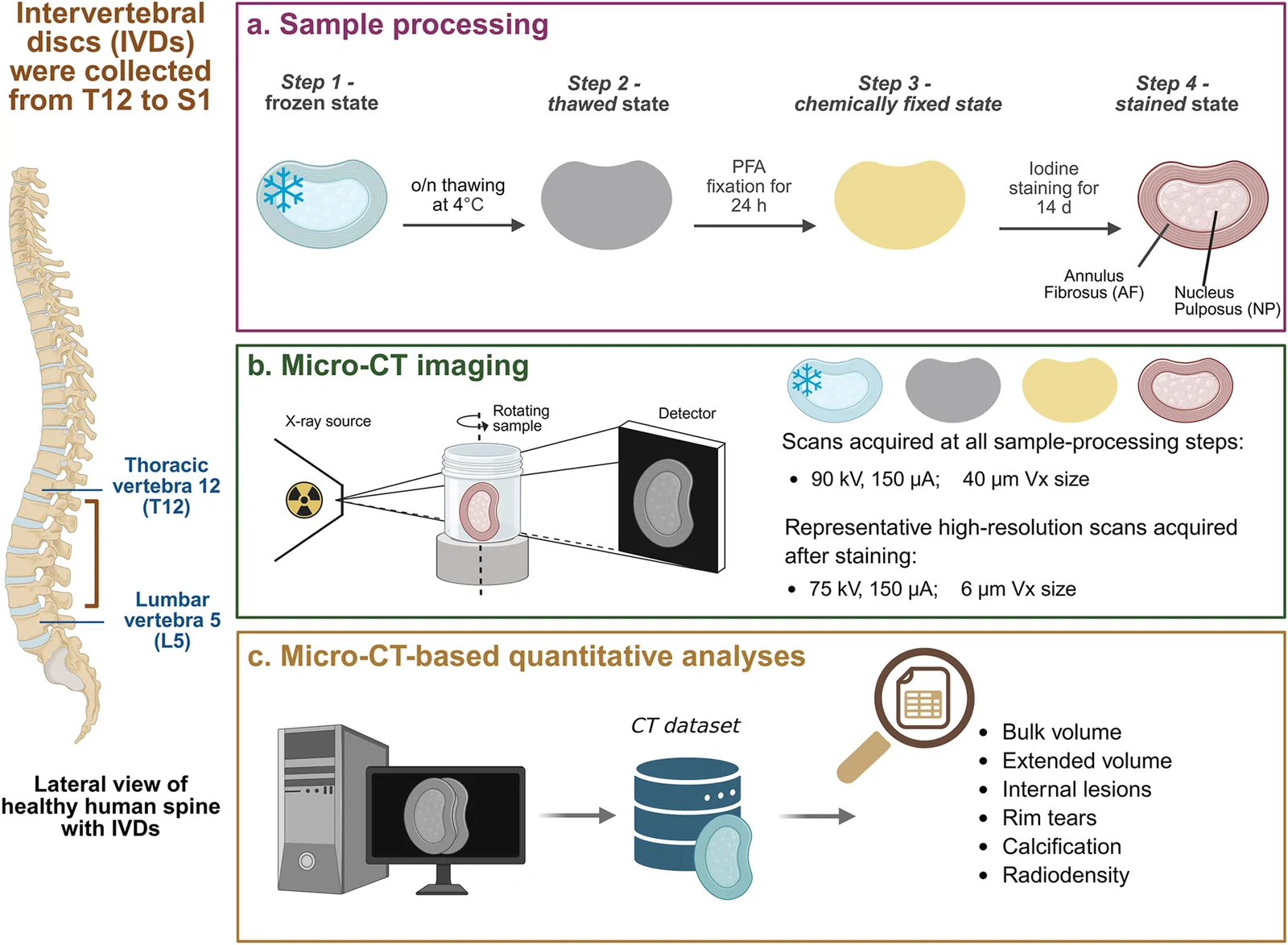
**Schematic illustration of the materials and methods applied in this study.** (**a**) Sample processing steps. (**b**) Micro-CT imaging analysis. (**c**) Volume analysis and radiodensity measurement.

Fifteen IVDs from seven human cadaver bodies were provided by Science Care (Phoenix, AZ, USA). Samples were accessed for research purposes between March 2022 and August 2023. The donors were four females and four males with a mean age of 59.6 (range, 26–75 years) and mean Body mass index (BMI) of 30.1 (range, 20.6–54.9) donor information summarized in Table 1. After thawing at room temperature, the posterior vertebral arch is separated from the vertebral body by cutting the connecting pedicles with an oscillating saw. Then, for each selected segment, the adjacent vertebral bodies are separated by transecting the interposed discs. The trabecular portions of the two vertebral bodies adjacent to the target disc are removed by cutting as close as possible to the endplates while staying superficial to the cortical shell to avoid damaging the disc. Finally, the remaining trabecular bone and CEPs were removed with a bone rongeur and a scalpel. The analyzed discs were collected from thoracic 12 (T12) to lumbar 5 (L5) regions of the spine. Sample processing involved several steps (Fig. 1a): IVDs were collected from already frozen spines at −20 °C (*step 1* – frozen state), then they were subsequently thawed at 4 °C overnight (o/n) (*step 2* – thawed state), chemically fixed in 4% paraformaldehyde for 24 h (*step 3* – chemically-fixed state), and finally stained in an aqueous solution of 3.75% Lugol’s iodine (1.25% w/v of I_2_ and 2.5% w/v of KI) for 14 days (*step 4* – stained state). The discs were kept in the same staining solution at room temperature throughout the staining period. Afterwards, the discs were rinsed in distilled water for 2 days.

**Table 1:** Characteristics of the donor population included in the study.

Patient number	Tested levels (*N* = 15)	Sex	Age	Height [cm]	Weight [kg]	BMI [Kg/m2]
1	T12-L1	Male	75	178	68	21.5
2	L4-L5	Male	63	173	82	27.4
3	T12L1, L2L3, L4L5	Male	52	180.3	73.5	22.6
4	T12L1, L2L3, L4L5	Female	61	157.5	91.6	36.9
5	T12L1, L2L3, L4L5	Female	68	165.1	149.7	54.9
6	T12L1	Female	72	170.2	77.1	26.6
7	T12L1, L2L3, L4L5	Female	26	162.6	54.4	20.6

### 2.2 Ethics declaration

The use of the human cadaveric material was performed according to the Swiss Federal Act on Research involving Human Beings n.810.30 (Human Research Act, HRA, of The Federal Assembly of the Swiss Confederation) of 30 September 2011 and the Guidelines of the Swiss Academy of Medical Sciences, updated 2014. Donors have formally agreed on the use of body parts for research by signing written informed consent forms. The experimental protocols have been approved by the Kantonale Ethikkommission Zürich (BASEC-Nr. 2020-02330) on the 1^st^ October 2020 and Zürich (BASEC-Nr. 2022-01966) on the 2^nd^ December 2022.

### 2.3 Micro-CT imaging

Micro-CT analysis was carried out using an EasyTom XL Ultra 230–160 micro/nano-CT scanner (RX Solutions, Chavanod, France). The specimens were analyzed at all processing steps, starting from the frozen state, followed by thawing, formalin fixation, and ultimately after tissue staining, with the same acquisition parameters. Sample holders were custom-made from polystyrene to stabilize the IVD during scanning and minimize unwanted movement while avoiding excessive compression of the disc. The scanner operated at 90 kV and 150 μA with a nominal resolution of 40 μm voxel (Vx) size (Fig. 1b). The samples were scanned over 360° with a rotation step of 0.31° and a frame average of 10 resulting in a total scan time of 42 min. All the CT datasets were reconstructed using the filtered back-projection algorithm, a small ring artefact reduction and a 75% sine window function. In specific regions of interest, high-resolution micro-CT zoom scans were acquired for the stained samples at 75 kV and 150 μA with a nominal resolution of 6 μm Vx size, a rotation step of 0.25° and a frame average of 5 for a total scan time of about 1 h 20 min. All micro-CT scans were carried out in a temperature-controlled environment at 24 °C.

The CT datasets were analyzed using the software application Avizo (Thermo Fisher Scientific, MA, USA). For each sample processing step, the volumes of the entire sample, lesions (e.g. internal lesions and rim tears), and calcifications were defined (Fig. 1c). The whole disc was first segmented using the Otsu algorithm [25], providing an initial global mask of the specimen. Starting from this segmentation, two derived masks were generated: a) the *bulk volume,* obtained by applying a 3D filling operation to the Otsu mask, and b) the *extended volume,* obtained by applying a 2D filling operation performed slice-by-slice along the axial plane. For lesion and calcification segmentation, global thresholds were applied to the disc volumes for each stage of sample processing. Lesions consisted of all pixels with grayscale intensity values ranging from 0 to one unit below the previously determined Otsu threshold (range: 0 to (Otsu −1)). Open rim tears were quantified as the difference between the void volume identified in the extended volume mask and the internal lesion volume. Calcifications were segmented from 16-bit TIFF images using global thresholding across the frozen, thawed, and chemically fixed conditions. IVD height was quantified from the bulk volume previously used for volumetric analysis. After aligning the disc axial direction with the z-axis, a spatially resolved height map was generated through column-wise integration of the binary mask. Local height was calculated at each in-plane position, corresponding to a sampling area of 2.56 × 10^−2^ mm^2^. Maximum and mean local heights were used as summary measures.

### 2.4 Radiodensity measurements

The reconstructed micro-CT volumes were analyzed in terms of radiodensity by converting reconstructed gray values to Hounsfield Units (HU) using an empirical calibration. HU were reported as water/air-referenced (HU-equivalent) values to enable quantitative comparison across samples and processing stages. Distilled water (0 HU) and air (−1000 HU) were scanned under identical acquisition and reconstruction parameters, and gray values were mapped to HU according to the following equation (Eq. (1)):

Eq.1 $$Hounsfield\;Unit=\frac{\left(GV_{tissue}-GV_{water}\right)}{\left(GV_{water}-GV_{air}\right)}\times 1000$$

where GV_tissue_, GV_water_ and GV_air_ are the mean reconstructed gray values measured in the tissue, distilled water, and air, respectively.

Radiodensity analysis was conducted for the frozen and iodine-stained states, as these conditions provided the highest microstructural contrast (Fig. 1c).

### 2.5 Statistical analysis

A linear mixed effect model was employed to assess the effect of sample preparation on the measured volumes, considering the preparation steps as fixed effects and sample name as random effect. The thawed state was selected as the reference level, as it represents the condition most similar to the native tissue state. To further characterize sources of baseline variability, alternative random-effects structures were evaluated and compared. In addition to a model including sample name as the sole random effect, models with crossed random effects were fitted in which donor (spine ID) and spinal segment were specified as independent random intercepts (model B). The crossed formulation allows to attribute the variance to donor specific or anatomical level specific differences. BMI category was included as an additional random effect to evaluate its contribution to baseline variance (model C). The described mixed effect models were applied to height and disc volume, calcification volume, and log-transformed volumes of internal lesion and rim tears, to account for the skewed distribution of the data.

## 3 Results

### 3.1 Volumetric analyses

**Fig. 2:**
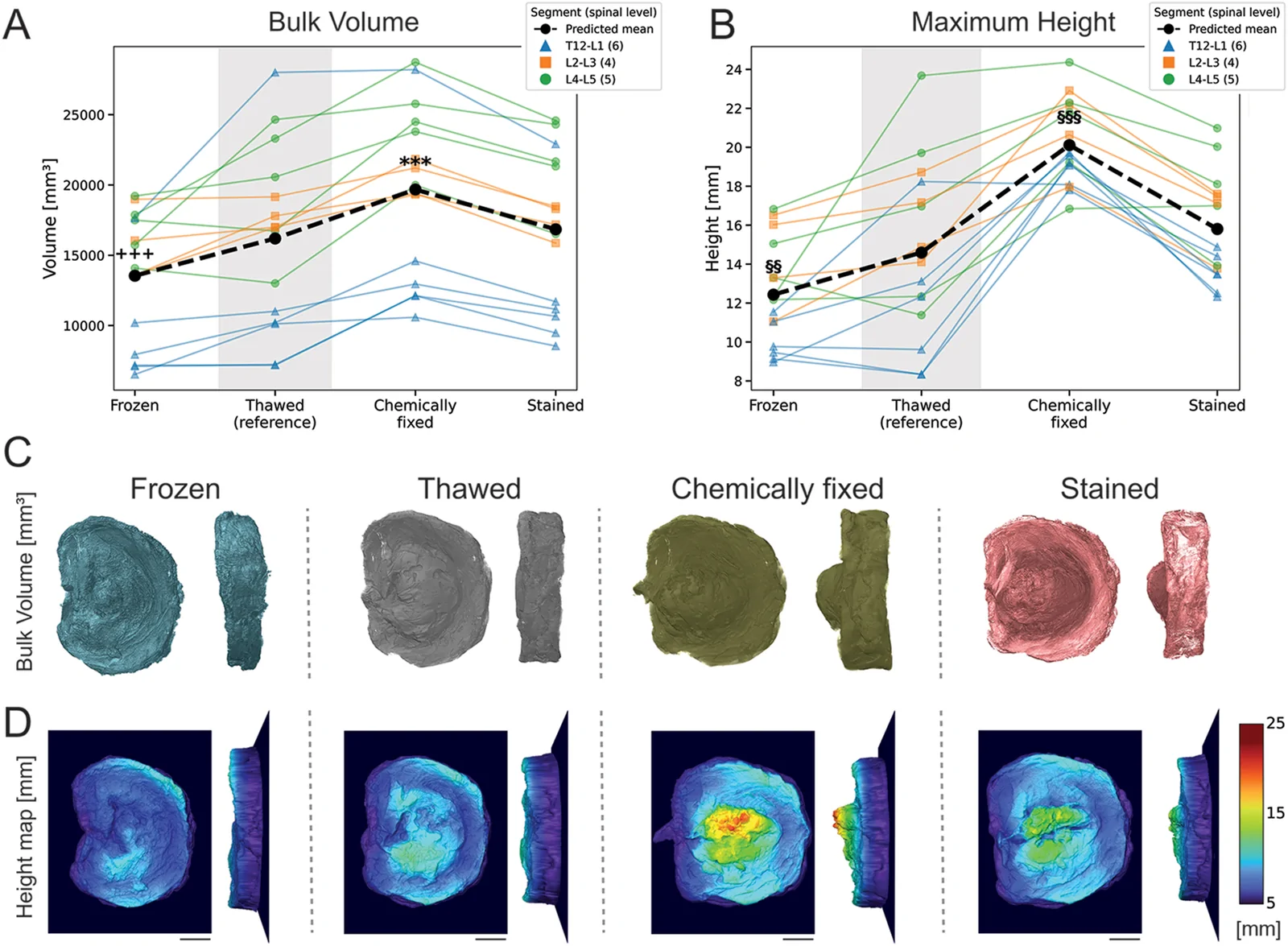
**Volumetric changes** of **intervertebral discs throughout the sample processing steps. a)** Bulk volume values measured across processing conditions. Colored trajectories connect repeated measurements from the same specimen; marker shape indicates the spinal segment (see legend). The black dashed line represents the predicted mean obtained from the mixed effect model. Statistically significant differences are represented for each comparison as follows: +++ *p* = 4.01 × 10^−5^, ∗∗∗*p* = 6.86 × 10-8. (**b**) Maximum height values measured across processing conditions. Colored trajectories connect repeated measurements from the same specimen; color identifies the specimen and marker shape indicates the spinal segment (see legend). The black dashed line represents the predicted mean obtained from the mixed effect model. Statistically significant differences are represented for each comparison as follows: §§*p* = 0.0019, §§§ *p* = 2.4 × 10^−15^. **(c)** Representative 3D volume renderings of the IVDs during sample processing. Scale bars: 10 mm (Voxel size = 40 μm). **(d)** Representative height maps renderings of the IVDs during sample processing. Scale bars: 10 mm (Voxel size = 40 μm) Colormap: 5-25 mm.

The bulk volumes of the samples varied significantly across processing conditions (Fig. 2a, c). Using the thawed state as reference, the frozen condition showed a significant reduction in volume (−16.4%, *p* = 0.0000401). Formalin fixation caused a statistically significant volume increase (+21.5%, *p* = 6.86 × 10^−8^) compared to the thawed state, with pronounced swelling of the NP (Fig. 2c). Subsequent iodine staining resulted in volume decrease relative to the formalin fixed state. The iodine induced shrinkage partially compensates for fixation-induced swelling, so the resulting volume is not significantly different from thawed state (Fig. 2a). Including spinal segment and BMI as crossed random effects revealed that spinal segment accounted for 46.5% of the total variance, whereas BMI contributed negligibly (4.1%).

### 3.2 Height analyses

Maximum IVD height varied across processing conditions (Fig. 2b, d). Using the thawed state as reference, freezing was associated with a significant reduction in maximum height (−14.8%, *p* = 0.0019), whereas formalin fixation resulted in a marked and statistically significant increase (+37.8%, *p* = 2.4 × 10^−15^). Lugol staining was associated with a modest, non-significant increase relative to the thawed condition (+8.3%, *p* = 0.082) (Fig. 2b). Including spinal segment and BMI as crossed random effects revealed that spinal segment accounted for 41.7% of the total variance, whereas BMI accounted for 14.5%. Analysis of mean rather than maximum disc height showed the same overall pattern, although the magnitude of the changes was smaller: freezing was associated with a reduction in mean disc height (−7.7%, *p* = 0.0033), formalin fixation with an increase (+15.9%, *p* = 1.2 × 10^−9^), and Lugol staining with a non-significant increase (+4.5%, *p* = 0.084).

### 3.3 Internal lesions and rim tears analyses

**Fig. 3:**
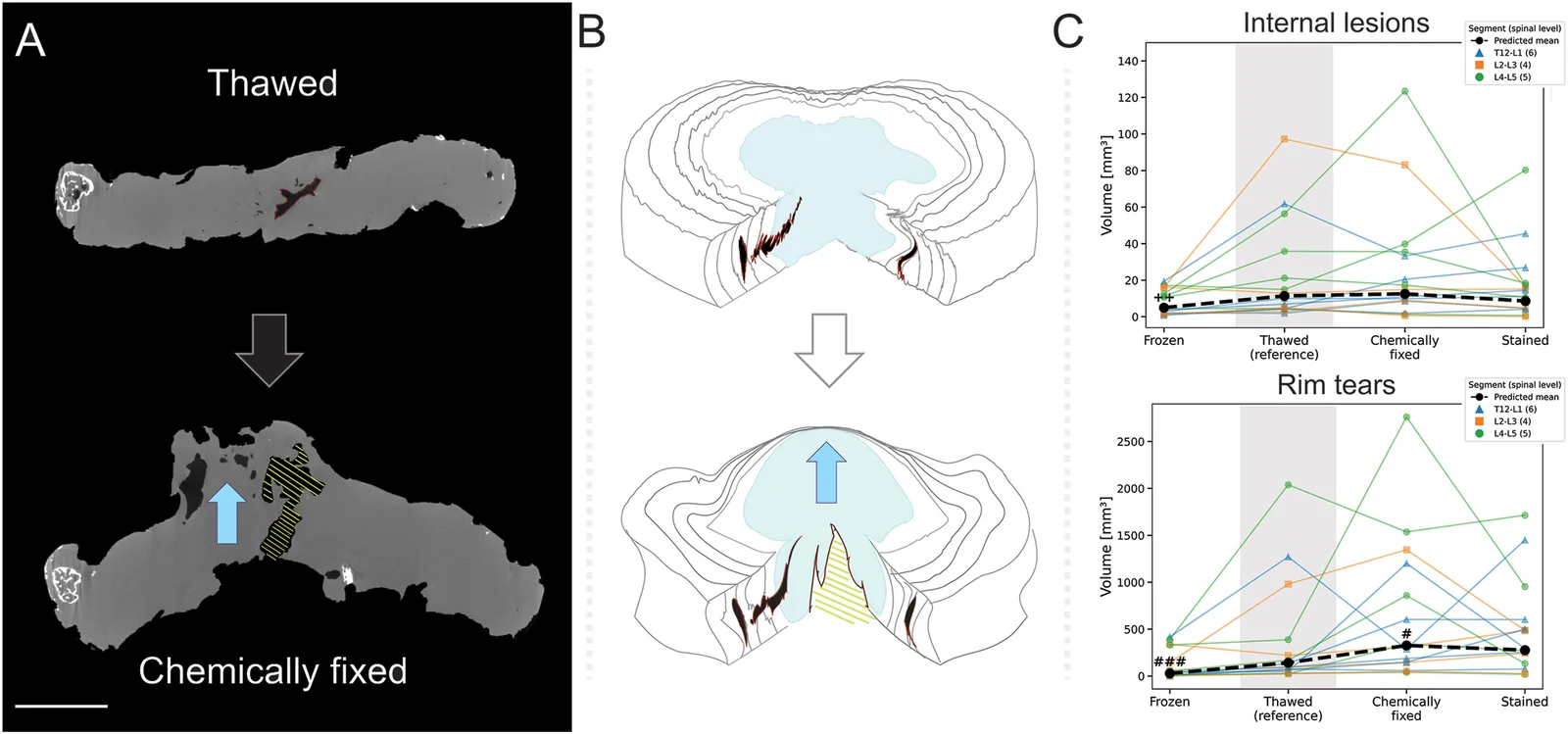
**Internal lesions and rim tear measurements of intervertebral discs throughout the sample processing steps. a)** Representative axial micro-CT sections of the IVDs comparing the thawed and chemically fixed states. Internal lesion is outlined in dark red, rim tears are highlighted with yellow stripe pattern, and NP bulging is marked with an arrow. (Scale bar = 10 mm Voxel size = 40 μm) **b)** Schematic drawings illustrating the internal lesions, the disc deformation and the increase in rim tears. Internal lesions are outlined in dark red, rim tears are indicated by striped pattern, and NP bulging is marked with an arrow. **c)** Volumes of internal lesions and rim tears in mm^3^ across processing conditions. Colored trajectories connect repeated measurements from the same specimen; marker shape indicates spinal level (see legend). The predicted mean values from the mixed effect model are represented in black and connected across condition through a dashed line. Statistically significant differences are represented for each comparison as follows: ++ *p* = 0.0039, ###*p* = 0.000016, #*p* = 0.0239.

Lesions were quantified for each sample processing step, and the resulting values are displayed in Fig. 3. The internal lesions were significantly affected by the freezing process, causing a clear and significant reduction in the volume (−56.6%, *p* = 0.0039). No significant volume variation from the baseline measured at the thawed state was identified in the other processing steps (Fig. 3c).

Rim-tear volume was lowest in the frozen state, showing a significant reduction relative to the thawed condition (−79.8%, *p* = 0.000016). In contrast, formalin fixation caused a significant increase in open lesion volume relative to the reference state (+131.1%, *p* = 0.0239) (Fig. 3c). In contrast to bulk volume, internal lesions and rim tears volumes showed only a limited dependence on spinal level when considered as additional random effect, accounting for less than 7.3% and 7.0% of the variance, respectively. For both measures, the dominant source of variability was inter-specimen differences, explaining 34.3% and 57.0% of the variance across models. Inclusion of BMI contributed negligibly (0.0%) to internal and open lesion variability.

### 3.4 Calcification analyses

**Fig. 4:**
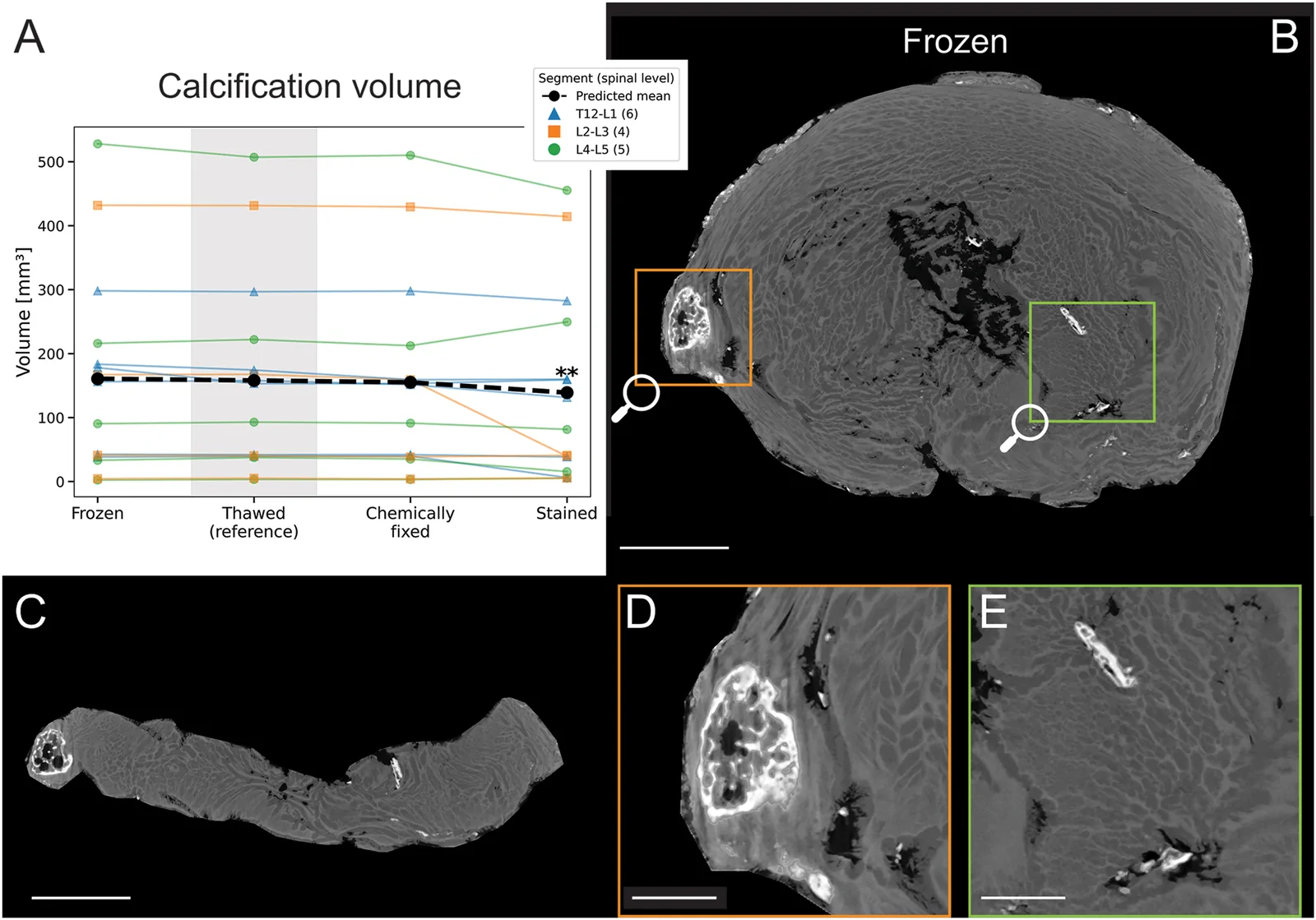
**Calcification measurement of intervertebral discs throughout the sample processing steps. a)** Calcification volumes measured across processing conditions. Colored trajectories connect repeated measurements from the same specimen; marker shape indicates spinal level (see legend). The predicted mean values from the mixed effect model are represented in black and connected across condition through a dashed line. Statistically significant differences are represented for each comparison as follows: ∗∗*p* = 0.0044 **b)** Representative axial micro-CT section showing calcified regions (highlighted boxes) Scale bar: 10 mm (voxel size: 40 μm). **c)** Sagittal reconstruction of the IVD displaying the spatial distribution of calcifications. Scale bar: 10 mm (voxel size: 40 μm). **(d, e)** Magnified views of peripheral **(d)** and internal **(e)** calcifications. Scale bars: 0.5 mm (voxel size: 40 μm).

Calcification volume remained unchanged after freezing and formalin fixation compared with the thawed condition (Fig. 4). Iodine based staining reduced the calcification volume (−12.1%, *p* = 0.004474) when compared to the thawed state. Calcification volume showed a limited dependence on spinal level, accounting for 8.9% of the total variance while inter specimen variability explained most of the variance (79.0%). When BMI category was included as a crossed random effect, it accounted negligibly (0.0%).

### 3.5 Radiodensity measurement of the intervertebral disc

**Fig. 5:**
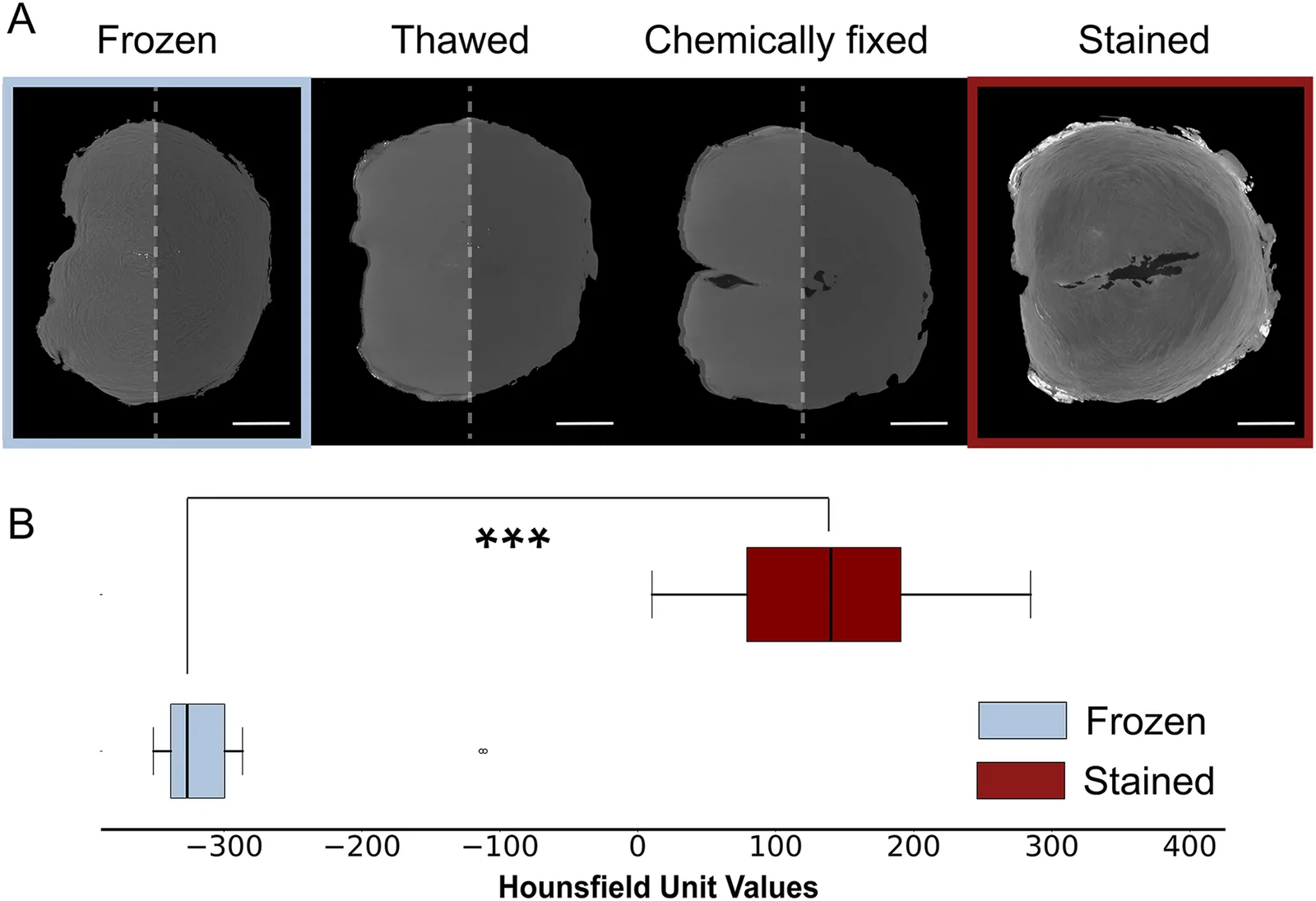
**Radiodensity measurements of intervertebral discs. a**) Representative axial micro-CT sections of the IVD across processing conditions. For each condition, the left half is displayed using condition-specific windowing to optimize visualization, whereas the right half is displayed using identical windowing parameters defined on the iodine-stained dataset (Lugol-derived WL/WW), enabling direct visual comparison across conditions. Scale bars: 10 mm (Voxel size = 40 μm). **b)** Distribution of HU values of the IVDs in the frozen and stained states. Boxes indicate median and interquartile range; whiskers indicate range. ∗∗∗*p* = 2.12 × 10^−13^.

Radiodensity differed markedly between conditions (Fig. 5). Under Lugol-derived windowing, frozen, thawed and chemically fixed datasets exhibited minimal internal contrast, whereas iodine staining shifted signal intensities to a higher range and enhanced structural definition. Quantitatively, median radiodensity increased from −326.74 HU in the frozen state to 140.06 HU in the stained one (*p* = 2.12 × 10^−13^).

### 3.6 High-resolution zoom scans

**Fig. 6:**
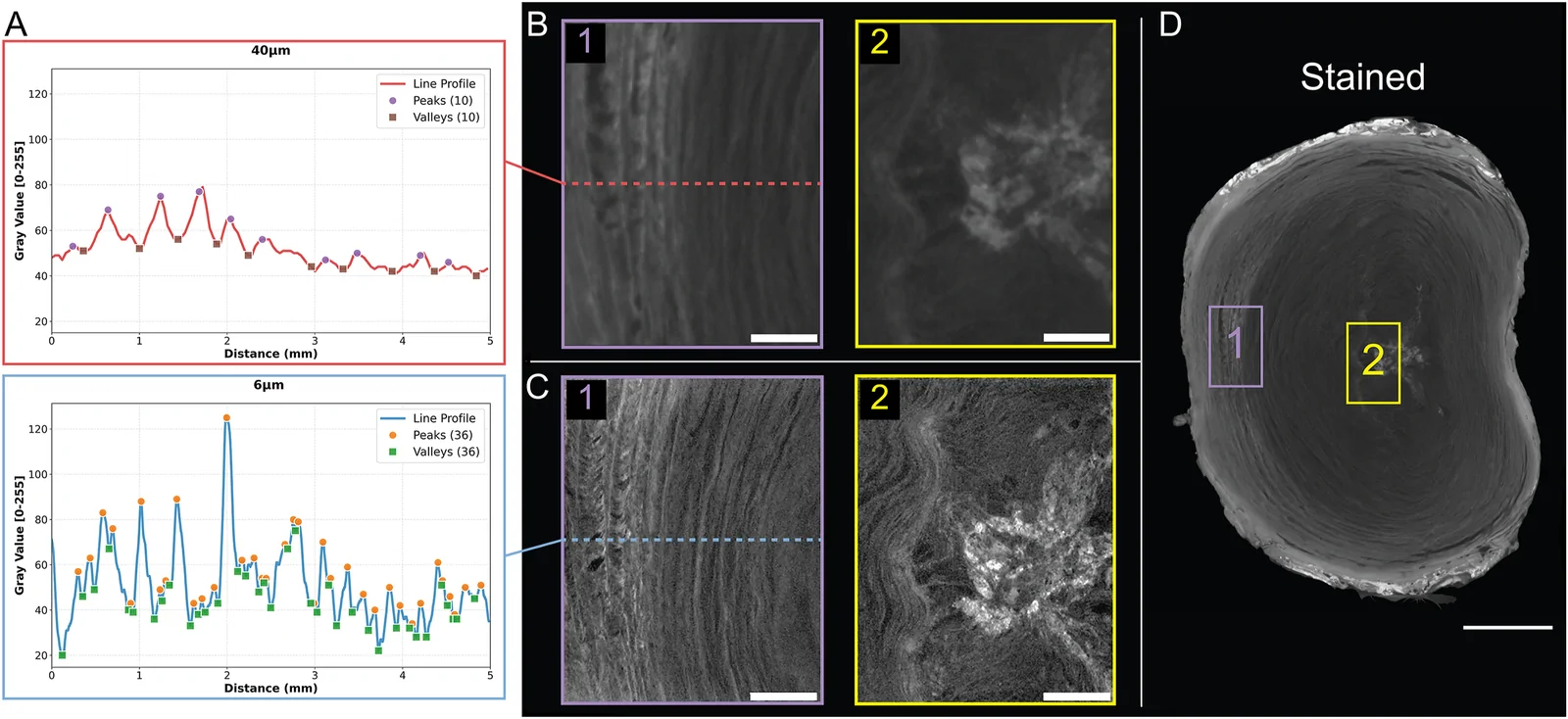
**High-resolution imaging enabled by fixation and iodine staining. a)** Intensity profiles extracted along representative lines at standard resolution (voxel size: 40 μm) and high resolution (voxel size: 6 μm), showing increased signal variability and feature definition at higher resolution. **b)** Enlarged views of selected regions acquired at standard resolution (40 μm voxel size). **c)** Corresponding high-resolution zoom scan (6 μm voxel size) revealing fine internal microstructural features. **d)** Axial microCT section of the stained sample at standard resolution (40 μm voxel size) indicating the regions (boxes 1 and 2) from which enlargements and high-resolution zoom scans were obtained. Scale bars: 10 mm **(d)**, 2 mm **(b,c)**.

The frozen and iodine-stained states provided clear structural contrast within the IVD, enabling visualization of internal architecture, whereas the thawed and chemically fixed conditions showed nearly homogeneous radiodensity and loss of internal structural definition. Frozen samples could only be acquired using standard-resolution scans, as prolonged acquisitions produced motion artifacts associated with progressive thawing. Therefore, high-resolution zoom scans were acquired from selected representative regions of interest in the iodine-stained state, with formalin fixation ensuring structural stability at room temperature during acquisition. These scans revealed fine internal microstructural features not discernible in standard-resolution datasets (Fig. 6).

## 4 Discussion

Contrast-enhanced micro-CT of the human IVD is strongly influenced by sample preparation. The present study shows that freezing, fixation and iodine staining introduce distinct and quantifiable alterations in tissue volume, morphology and radiodensity, which must be considered when interpreting structural features in soft-tissue micro-CT datasets. By imaging the same sample across processing states, we quantified changes in volume, height, radiodensity, calcification, lesions, and processing-induced deformations, providing a framework for optimizing imaging workflows and interpreting preparation artifacts. Previous studies have utilized laboratory-based X-ray imaging to visualize deep frozen ovine vertebral segments [26], as freezing is a conventional preservation method for spinal specimens that allows further biomechanical testing without being affected by the freezing-thawing cycle [27]. Similarly to animal samples, in human IVDs, biomechanical properties such as elastic and creep responses have been preserved during frozen storage [28]. When analyzing the effect of freezing in animal and human soft tissues, previous work reported changes in CT attenuation and volume after freezing, with some tissues experiencing increases in volume while others showed shrinkage [29]. Consistent with this, our study observed a reduction in sample volume in the frozen state compared to the thawed state. This can be attributed to the excellent swelling capacity of the IVD, which is largely due to its high proteoglycan content. Water within the IVD is stored as either bound water, associated with protein structures such as collagen fibers, or as free water, which interacts more with proteoglycans [30]. Previous studies have shown that IVD tissue can increase in volume by more than 50% [31,32]. However, during the freezing process, some of the free water may be lost, as it is not retained within the tissue polymeric network, contributing to the observed volume reduction. Despite this volumetric contraction, frozen samples preserved recognizable lamellar architecture, allowing structural assessment without chemical alteration, due to the dehydration enhancement effects. However, progressive thawing during scanning introduced motion artifacts that restricted acquisition time and prevented high-resolution imaging.

In contrast, formalin fixation induced a significant increase in the average sample volume by 21.5% when compared with the thawed state. This volume expansion is associated with the occurrence of external deformation of the tissue caused principally by the swelling of the NP as also highlighted by the significant increase in rim tears volume. Similar deformations have been observed macroscopically in bovine and sheep IVDs [24,33]. Both studies reported swelling of the NP and separation of the annulus lamellae following chemical fixation. A similar trend in volume increase within the first 4 days of immersion in buffered formalin solution has been quantified for other soft tissues such as skeletal muscle, cardiac muscle, and cerebellum [34], reflecting osmotic imbalance and cross-linking-induced matrix relaxation. Nevertheless, formalin remains essential for long-term preservation and stabilization of spinal disc tissue architecture, maintaining the amount and degree of elastin and collagen cross-linking [35].

Following fixation, iodine staining was employed in this study to enhance the X-ray contrast and visualize the microstructure of non-mineralized IVD components The high diffusivity and ease of preparation of Lugol’s solution make it suitable for staining relatively large samples [20,[36], [37], [38]]. Consistent with the work of Disney et al. [24], full staining of human IVDs required two weeks. Among commonly used contrast agents, PMA and Phosphotungstic Acid provide strong soft-tissue contrast but produced a pronounced diffusion front and incomplete penetration in large bovine IVD specimens [24]. Their relatively large polyoxometalate structures may limit diffusion through dense, avascular fibrocartilaginous tissues because of steric hindrance and interactions with extracellular matrix components. By contrast, the more mobile iodine species present in Lugol’s solution can provide faster and more homogeneous penetration, making this agent suitable for visualization of the overall AF-NP morphology. Iodine-based agents have also been used successfully for micro-CT imaging of rat and mouse IVDs and of meniscal tissue, which has a similarly dense collagenous architecture [19,24,39]. After staining, the samples shrank compared to the chemically fixed state, bringing volumes closer to thawed-state values but with deformation inherited from prior fixation. Shrinkage associated with iodine staining has been documented by other studies as well [24,34], and to minimize this effect, the lowest concentration necessary for adequate contrast was employed. A substantial proportion of volume variability across all processing stages was explained by spinal level, whereas BMI contributed negligibly. This indicates that anatomical position represents the dominant source of volumetric heterogeneity in the IVD and must be accounted for when comparing preparation-induced changes. This reflects the known cranio-caudal scaling of load-bearing capacity in the spine.

Notably, iodine staining increased the radiodensity of the IVDs by 148.6% compared to the frozen stage. The contrast enhancement shifted attenuation values into a higher dynamic range, enabling visualization of internal disc structures and facilitating segmentation of non-mineralized components. Importantly, staining preserved the deformation inherited from fixation, indicating that contrast preparation improves visibility but does not restore native geometry. Stabilization through fixation and staining enabled high-resolution zoom imaging, revealing fine microstructural features.

Furthermore, the intensity profiles in Fig. 6 quantitatively demonstrate the resolution-dependent detectability of annular lamellar periodicity. At 6 μm voxel size, a markedly higher density of attenuation peaks is observed, particularly towards the inner annulus where lamellae are thinner and more densely packed. These oscillations reflect transitions between individual lamellae and interlamellar regions. In contrast, at 40 μm resolution, high-frequency structural components are attenuated by partial volume effects and modulation transfer limitations, causing multiple interfaces to merge into broader and less intense peaks. The higher-resolution sampling approaches the Nyquist requirement for typical lamellar spacing (∼0.05–0.5 mm) [40], thereby preserving native microstructural heterogeneity that remains spectrally suppressed at conventional voxel sizes.

Internal lesions were consistently detected across preparation stages, with stable relative volume after thawing, indicating that preparation primarily affect global geometry rather than intrinsic tissue discontinuities. Consequently, contrast-enhanced micro-CT appears suitable for three-dimensional, non-destructive quantification of disc lesions relevant to functional impairment [41].

The proximity and coexistence of fissures and calcifications within the IVD have been highlighted by histopathological studies [42]. This correlation may reflect increased local stress concentration around hard inclusions embedded in the softer disc matrix, potentially promoting tissue damage and rupture [42]. Calcified hard tissue can be properly visualized with micro-CT thanks to its intrinsic high radiodensity. In fact, calcifications were visible across processing steps, with no significant change in volumetric values between frozen, thawed and fixed conditions. However, iodine staining produced an apparent reduction in calcified volume. This decrease is unlikely to reflect mineral loss, as Lugol solution does not act as a decalcifying agent, but rather results from increased attenuation of surrounding soft tissues that reduces contrast between mineralized inclusions and the matrix. Consequently, small calcifications may fall below the segmentation threshold after staining.

### 4.1 Limitations

This study is not without limitations. Chemical fixation, while essential for preserving tissue and currently the conventional approach for histopathological evaluation of IVD, had the strongest impact on tissue morphology. In addition, the overall sample processing for contrast-enhanced micro-CT analysis is time-consuming, but the reached stability of the IVD tissue microstructure allows several additional targeted high-resolution zoom scans. Sample harvesting is a critical methodological step. We analyzed the AF and NP of isolated lumbar IVDs rather than intact IVD–endplate preparations. This reduced specimen size, facilitated contrast-agent penetration, and enabled a smaller field of view and higher spatial resolution. However, preserving the CEPs would maintain mechanical confinement and might reduce fixation-induced deformation. Because sectioning may introduce procedure-related damage, lesion measurements should be interpreted as relative processing-associated changes rather than as a diagnostic assessment of native pathology. The thawed state was used as the reference condition, incorporating any harvesting-related defects into the baseline lesion pattern and allowing the incremental effects of subsequent processing steps to be assessed. This reflects conventional histological workflows, in which tissue manipulation generally precedes fixation. The freezing protocol also warrants further optimization. Storage at approximately −20 °C or −80 °C may differentially affect disc mechanical properties [43], and the optimal conditions may depend on whether the primary objective is biomechanics, microstructural imaging, or long-term preservation. Frozen-state imaging also showed potential: in this study, it enabled visualization of calcifications and global structural patterns, while recent advances in deep-frozen and cryogenic contrast-enhanced micro-CT support high-resolution 3D analysis of soft tissues at sub-zero temperatures [26,44].

Future refinements in staining protocols, fixation procedures, and freezing methods may further minimize artifacts while preserving microstructural fidelity, identifying the ideal combination to limit and minimize preparation-induced effects and enable complementary histomorphological characterization.

### 4.2 Conclusions

In conclusion, the non-destructive examination of frozen, thawed, chemically fixed, and contrast-stained IVDs using micro-CT revealed the advantages and trade-offs of each processing stage. This study demonstrated 3D visualization of human IVDs in an *ex vivo* experimental setting. Iodine-enhanced high-resolution scans enabled detailed visualization of fine disc microstructures, whereas frozen-state imaging allowed gross assessment of AF lamellae with minimal chemical alteration. By quantitatively comparing volumetric changes, radiodensity, calcifications, lesions, and deformation at different processing stages, this study provides a reference for optimizing contrast-enhanced micro-CT workflows and distinguishing pathological features from preparation-induced artifacts in IVD research.

## Author contributions

**RB**: data curation, formal analysis, investigation, methodology, writing – original draft; **FO**: data curation, formal analysis, investigation, methodology, writing – original draft; **MF**: writing – review and editing; **SB**: statistical expertise; **DV**: writing – review and editing; **JW**: conceptualization, writing – review and editing; **AP**: conceptualization, methodology, investigation, supervision, writing – original draft, writing – review and editing. All authors have reviewed the manuscript and approved the final version.

## Conflict of interest

The authors declare no competing interests.

## References

[1] GBD 2021 Low Back Pain Collaborators (2023). Global, regional, and national burden of low back pain, 1990-2020, its attributable risk factors, and projections to 2050: a systematic analysis of the Global Burden of Disease Study 2021. Lancet Rheumatol..

[2] Manchikanti L., Singh V., Falco F.J., Benyamin R.M., Hirsch J.A. (2014). Epidemiology of low back pain in adults. Neuromodulation.

[3] Teraguchi M., Yoshimura N., Hashizume H., Muraki S., Yamada H., Minamide A. (2014). Prevalence and distribution of intervertebral disc degeneration over the entire spine in a population-based cohort: the Wakayama Spine Study. Osteoarthr. Cartil..

[4] Raj P.P. (2008). Intervertebral disc: anatomy-physiology-pathophysiology-treatment. Pain Pract..

[5] Humzah M.D., Soames R.W. (1988). Human intervertebral disc: structure and function. Anat. Rec..

[6] Eyre D.R., Muir H. (1977). Quantitative analysis of types I and II collagens in human intervertebral discs at various ages. Biochim. Biophys. Acta.

[7] Smith L.J., Fazzalari N.L. (2009). The elastic fibre network of the human lumbar anulus fibrosus: architecture, mechanical function and potential role in the progression of intervertebral disc degeneration. Eur. Spine J..

[8] Johnstone B., Bayliss M.T. (1995). The large proteoglycans of the human intervertebral disc. Changes in their biosynthesis and structure with age, topography, and pathology. Spine.

[9] Whatley B.R., Wen X. (2012). Intervertebral disc (IVD): structure, degeneration, repair and regeneration. Mater. Sci. Eng. C.

[10] Luoma K., Riihimäki H., Luukkonen R., Raininko R., Viikari-Juntura E., Lamminen A. (2000). Low back pain in relation to lumbar disc degeneration. Spine.

[11] Sasiadek M.J., Bladowska J. (2012). Imaging of degenerative spine disease--the state of the art. Adv. Clin. Exp. Med..

[12] Leone A., Guglielmi G., Cassar-Pullicino V.N., Bonomo L. (2007). Lumbar intervertebral instability: a review. Radiology.

[13] Chiu A.P., Chia C., Arendt-Nielsen L., Curatolo M. (2024). Lumbar intervertebral disc degeneration in low back pain. Minerva Anestesiol..

[14] Colosimo C., Gaudino S., Alexandre A.M. (2011). Imaging in degenerative spine pathology. Acta Neurochir. Suppl..

[15] Holzapfel G.A., Schulze-Bauer C.a.J., Feigl G., Regitnig P. (2005). Single lamellar mechanics of the human lumbar anulus fibrosus. Biomech. Model. Mechanobiol..

[16] Marchand F., Ahmed A.M. (1990). Investigation of the laminate structure of lumbar disc anulus fibrosus. Spine.

[17] Adam C., Rouch P., Skalli W. (2015). Inter-lamellar shear resistance confers compressive stiffness in the intervertebral disc: an image-based modelling study on the bovine caudal disc. J. Biomech..

[18] Stein D., Assaf Y., Dar G., Cohen H., Slon V., Kedar E., Medlej B., Abbas J., Hay O., Barazany D., Hershkovitz I. (2021). 3D virtual reconstruction and quantitative assessment of the human intervertebral disc’s annulus fibrosus: a DTI tractography study. Sci. Rep..

[19] Orellana F., Grassi A., Nuss K.M., Wahl P., Neels A., Zaffagnini S., Parrilli A. (2024). Spatial and temporal evaluation of iodine uptake and radiodensity in meniscus tissue using contrast-enhanced micro-CT. Heliyon.

[20] Zuncheddu D., Della Bella E., Schwab A., Petta D., Rocchitta G., Generelli S., Kurth F., Parrilli A., Verrier S., Rau J.V., Fosca M., Maioli M., Serra P.A., Alini M., Redl H., Grad S., Basoli V. (2021). Quality control methods in musculoskeletal tissue engineering: from imaging to biosensors. Bone Res..

[21] Parrilli A., Grassi A., Orellana F., Lolli R., Marchiori G., Berni M., Fini M., Lopomo N.F., Zaffagnini S. (2024). 3D visualization of the human anterior cruciate ligament combining micro-CT and histological analysis. Surg. Radiol. Anat..

[22] Orellana F., Grassi A., Hlushchuk R., Wahl P., Nuss K.M., Neels A., Zaffagnini S., Parrilli A. (2024). Revealing the complexity of meniscus microvasculature through 3D visualization and analysis. Sci. Rep..

[23] Bhalla S., Lin K.H., Tang S.Y. (2017). Postnatal development of the Murine Notochord remnants quantified by high-resolution contrast-enhanced MicroCT. Sci. Rep..

[24] Disney C.M., Madi K., Bodey A.J., Lee P.D., Hoyland J.A., Sherratt M.J. (2017). Visualising the 3D microstructure of stained and native intervertebral discs using X-ray microtomography. Sci. Rep..

[25] Otsu N. (1979). A threshold selection method from gray-level histograms. IEEE Trans. Syst. Man Cybern..

[26] Kampschulte M., Erdmann G., Sender J., Martels G., Böcker W., ElKhassawna T. (2015). The development and validation of micro-CT of large deep frozen specimens. Scanning.

[27] Gleizes V., Viguier E., Féron J.M., Canivet S., Lavaste F. (1998). Effects of freezing on the biomechanics of the intervertebral disc. Surg. Radiol. Anat..

[28] Dhillon N., Bass E.C., Lotz J.C. (2001). Effect of frozen storage on the creep behavior of human intervertebral discs. Spine.

[29] Pech P., Bergström K., Rauschning W., Haughton V.M. (1987). Attenuation values, volume changes and artifacts in tissue due to freezing. Acta Radiol..

[30] Żak M., Pezowicz C. (2016). Analysis of the impact of the course of hydration on the mechanical properties of the annulus fibrosus of the intervertebral disc. Eur. Spine J..

[31] Bezci S.E., Nandy A., O’Connell G.D. (2015). Effect of hydration on healthy intervertebral disk mechanical stiffness. J. Biomech. Eng..

[32] Yang B., O’Connell G.D. (2019). Intervertebral disc swelling maintains strain homeostasis throughout the annulus fibrosus: a finite element analysis of healthy and degenerated discs. Acta Biomater..

[33] Melrose J., Smith S.M., Smith M.M., Little C.B. (2008). The use of Histochoice for histological examination of articular and growth plate cartilages, intervertebral disc and meniscus. Biotech. Histochem..

[34] Vickerton P., Jarvis J., Jeffery N. (2013). Concentration-dependent specimen shrinkage in iodine-enhanced microCT. J. Anat..

[35] Tan C., Dunn S., Kent G.N., Randall A.G., Edmondston S.J., Singer K. (2002). Does formalin-fixation alter the extent of collagen and elastin crosslinks in human spinal invertebral discs and ligamentum flavum?. J. Musculoskelet. Res..

[36] Pauwels E., Van Loo D., Cornillie P., Brabant L., Van Hoorebeke L. (2013). An exploratory study of contrast agents for soft tissue visualization by means of high resolution X-ray computed tomography imaging. J. Microsc..

[37] Gignac P.M., Kley N.J. (2014). Iodine-enhanced micro-CT imaging: methodological refinements for the study of the soft-tissue anatomy of post-embryonic vertebrates. J. Exp. Zool. B Mol. Dev. Evol..

[38] Jeffery N.S., Stephenson R.S., Gallagher J.A., Jarvis J.C., Cox P.G. (2011). Micro-computed tomography with iodine staining resolves the arrangement of muscle fibres. J. Biomech..

[39] Buckles M.M., Nur A., Warda N.H., Moncher J.C., Yunus Ansari M. (2025). High-resolution 3-Dimensional Micro-CT imaging of intervertebral discs using a novel contrast agent. JOR Spine.

[40] Newell N., Little J.P., Christou A., Adams M.A., Adam C.J., Masouros S.D. (2017). Biomechanics of the human intervertebral disc: a review of testing techniques and results. J. Mech. Behav. Biomed. Mater..

[41] Adams M.A., Roughley P.J. (2006). What is intervertebral disc degeneration, and what causes it?. Spine.

[42] Zehra U., Tryfonidou M., Iatridis J.C., Illien-Jünger S., Mwale F., Samartzis D. (2022). Mechanisms and clinical implications of intervertebral disc calcification. Nat. Rev. Rheumatol..

[43] Azarnoosh M., Stoffel M., Quack V., Betsch M., Rath B., Tingart M. (2017). A comparative study of mechanical properties of fresh and frozen-thawed porcine intervertebral discs in a bioreactor environment. J. Mech. Behav. Biomed. Mater..

[44] Maes A., Pestiaux C., Marino A., Balcaen T., Leyssens L., Vangrunderbeeck S. (2022). Cryogenic contrast-enhanced microCT enables nondestructive 3D quantitative histopathology of soft biological tissues. Nat. Commun..

